# Dataset of above and below ground traits assessed in Durum wheat cultivars grown under Mediterranean environments differing in water and temperature conditions

**DOI:** 10.1016/j.dib.2021.107754

**Published:** 2021-12-24

**Authors:** Fatima Zahra Rezzouk, Adrian Gracia-Romero, Shawn C. Kefauver, Maria Teresa Nieto-Taladriz, Maria Dolores Serret, José Luis Araus

**Affiliations:** a Integrative Crop Ecophysiology Group, Plant Physiology Section, Faculty of Biology, University of Barcelona, 08028 Barcelona, Spain; b AGROTECNIO (Center for Research in Agrotechnology), Av. Rovira Roure 191, 25198 Lleida, Spain; c Instituto Nacional de Investigación y Tecnología Agraria y Alimentaria (INIA), Ctra. de La Coruña Km. 7,5, 28040 Madrid, Spain

**Keywords:** Stable isotopes, Root traits, Leaf pigments, Canopy temperature, CT, canopy temperature, DTH, days to heading, HI, harvest index, ILP, irrigated late planting, INP, irrigated normal planting, GN, grain number, GNY, total grain nitrogen yield, GY, grain yield, NBI, nitrogen balance index, NDVI, normalized difference vegetation index, PCA, principle component analysis, PH, plant height, RA, root angle, RNP, rainfed normal planting, SRL, Specific root length, TGW, thousand grain weight, δ^13^C, carbon isotope composition, δ^15^N, nitrogen isotope composition, δ^18^O, oxygen isotope composition

## Abstract

Ideotypic characteristics of durum wheat associated with higher yield under different water and temperature regimes were studied under Mediterranean conditions. The focus of this paper is to provide raw and supplemental data from the research article entitled “Durum wheat ideotypes in Mediterranean environments differing in water and temperature conditions” [1], which aims to define specific durum wheat ideotypes according to their responses to different agronomic conditions. In this context, six modern (i.e. post green revolution) genotypes with contrasting yield performance (i.e. high vs low yield) were grown during two consecutive years under different treatments: (i) winter planting under support-irrigation conditions, (ii) winter planting under rainfed conditions, (iii) late planting under support-irrigation. Trials were conducted at the INIA station of Colmenar de Oreja (Madrid). Different traits were assessed to inform about water status (canopy temperature at anthesis and stable carbon isotope composition (δ^13^C) of the flag leaf and mature grains), root performance (root traits and the oxygen isotope composition (δ^18^O) in the stem base water), phenology (days from sowing to heading), nitrogen status/photosynthetic capacity (nitrogen content and stable isotope composition (δ^15^N) of the flag leaf and mature grain together with the pigment contents and the nitrogen balance index (NBI) of the flag leaf), crop growth (plant height (PH) and the normalized difference vegetation index (NDVI) at anthesis), grain yield and agronomic yield components. For most of the parameters assessed, data analysis demonstrated significant differences among genotypes within each treatment. The level of significance was determined using the Tukey-b test on independent samples, and ideotypes were modelled from the results of principle component analysis. The present data shed light on traits that help to define specific ideotype characteristics that confer genotypic adaptation to a wide range of agronomic conditions produced by variations in planting date, water conditions and season.

## Specifications Table

**Table:** 

Subject	Agronomy and Crop Science
Specific subject area	This dataset provides information on a wide range of traits that were used to characterize genotypic durum wheat ideotypes under different agronomic scenarios.
Type of data	Tables Figure
How data were acquired	Leaf pigments were assessed using a portable leaf-clip sensor (Dualex, Force-A, Orsay, France), which operates with a red reference beam at 650 nm and a UV light at 375 nm [2,3]. Canopy temperature (CT) was assessed using a portable infrared thermometer (PhotoTempTM MXSTM TD Raytek®, California; USA). The normalised difference vegetation index (NDVI) was measured from the ground using a GreenSeeker sensor (Trimble, Sunnyvale, CA, USA), and based on the contrasting reflectance of the canopy within the visible and near infrared regions of the spectrum. Root traits were obtained through a shovelomic approach [4], through the analysis of digitized root images using a Sony ILCE-QX1 camera (Sony Europe Limited, Brooklands; United Kingdom). The digital camera has a 20.1megapixel resolution, is equipped with a 23.2 mm × 15.4 mm sensor (type CMOS Exmor HD) and uses a 16 mm focal lens, and an exposure time of 1/60 s. RGB images were captured zenithally at 60 cm above the roots alongside a scale reference and saved in Tiff format for later analysis. Root angle (RA) was measured directly using a geometric protractor. Root RGB images were further analysed using GiaRoots (General Image Analysis of Roots, Georgia Tech Research Corporation and Duke University; USA), which is an open-source software for the automated analysis of root architecture [5]. Image processing was carried out using the adaptive image thresholding processing option,
	where around 200 images were computed per trial. The measured traits and the corresponding definition have been detailed previously in [5]. The stable carbon (δ^13^C) and nitrogen (δ^15^N) isotope composition of flag leaf and mature grains dry matter were assessed by grinding dried samples using a Mixer Mill (MM400, RETSCH GmbH, Germany) and subsampling approximately 1 mg of the material into tin capsules for further analysis using an elemental analyser (Flash 1112 EA; ThermoFinnigan, Schwerte, Germany) coupled with an isotope ratio mass spectrometer (Delta C IRMS, ThermoFinnigan), operating in continuous flow mode. The oxygen isotope composition (δ^18^O) of stem water was analysed using a cryogenic vacuum distillation line [6] followed by isotope-ratio infrared spectroscopy. Sample tubes were placed in a heated silicone oil bath (120 °C), and connected with Ultra-TorrTM unions (Swagelok Company, Solon, OH, USA) to a vacuum system (∼10−2 mbar), in series, with U-shaped collector tubes cooled with liquid N_2_. Ninety min after commencing extraction, the extracted xylem water was transferred into 2 ml vials and stored at 4 °C until analysis. Then δ^18^O of water was determined by isotope-ratio infrared spectroscopy using a Picarro L2120-I isotopic water analyser coupled to an A0211 high-precision vaporizer (Picarro Inc., Sunnyvale, CA, USA) [7].
Data format	Raw Analysed
Parameters for data collection	Days to heading (DTH) was assessed around heading, and measurements/sampling of leaf pigments, CT, NDVI, plant height (PH), flag leaf dry matter and stem-base water. At maturity, GY and yield components were assessed, and grains together with flag leaves were analysed for δ^13^C, δ^15^N and total N and C content. Further, the δ^18^O of stem water and root shovelomic traits were measured.
Description of data collection	DTH were determined during 2017-2018 only, when approximately 50 % of ears had emerged. PH was determined by placing a ruler zenithally in the central rows of each selected plot, values were then taken from the ground to the overall tip of the ears, excluding the awns. At maturity, ear density, grain number per ear (GN), thousand grain weight (TGW) and harvest index (HI) were assessed. Shovelomics were carried out in five random plants at maturity, which were dug manually from the first 15 cm of soil. δ^13^C, δ^15^N and total N and C content were analysed in dry matter of flag leaves at anthesis and in mature grains, and the δ^18^O of stem water was analysed at anthesis.
Data source location	Institution: Instituto Nacional de Investigación y Tecnología Agraria y Alimentaria (INIA) City: Madrid Country: Spain Latitude and longitude (and GPS coordinates) for collected samples/data: 40°04′N. 3°31′W. 590 m a.s.l.
Data accessibility	Repository name: Mendeley Data DOI: https://doi.org/10.17632/g86xjp5pbv.2 Direct URL to data: https://data.mendeley.com/datasets/g86xjp5pbv/2
Related research article	F. Z. Rezzouk, A. Gracia-Romero, S. C. Kefauver, M. T. Nieto-Taladriz, M. D. Serret, J. L. Araus. Durum wheat ideotypes in Mediterranean environments differing in water and temperature conditions. Agric. Water Manag., 259 (2022), 1-14. https://doi.org/10.1016/j.agwat.2021.107257

## Value of the Data

• The present data provides a reference to versatile physiological and agronomic plant traits collected above and below ground, with the aim of identifying potential durum wheat ideotypes under different growing scenarios.
• This dataset includes a wide spectrum of agronomic, morphological and physiological traits that can be potential proxies to understand the genotypic strategies of wheat to adapt to different growth conditions and give insights into more refined breeding programs.
• This dataset is also potentially useful for researchers involved in the agronomy of Mediterranean wheat. For this reason, a particular emphasis was given to secure variations in water regime and the growing temperature, alongside interannual variability. Overall, the range of
• The physiological traits evaluated are diverse, including above and below ground traits. Among the former, remote sensing traits evaluated at the canopy and leaf levels in conjunction with key lab traits such as the stable isotope signatures of the carbon and nitrogen stable isotopes in plant dry matter are included. The below-ground evaluation is even more noteworthy since it includes traits informing about root performance under field conditions: shovelomics and the oxygen isotope composition of the water at the base of the plant stem.
• The variety of growing conditions tested, as defined by the mean-yield trials, is very high, ranging up to twofold.

## 1 Data Description

Supplementary Table 1 displays averaged genotypic values of grain yield in a set of 24 durum wheat genotypes grown under different conditions, where means exhibiting different letters are significantly different (P < 0.05) according to the post-hoc Tukey-b test on independent samples within each treatment (irrigated normal planting (INP), rainfed normal planting (RNP) and irrigated late planting (ILP)). The experimental conditions of the field trials are given in Supplementary Table 2. The p values of the three-way (genotype, treatments and years) ANOVA are presented in Supplementary Table 3 for yield components, in Supplementary Table 4 for oxygen, carbon and nitrogen stable isotopes and N and C contents, and canopy temperature and leaf pigments, and in Supplementary Table 5 for root traits. Moreover, correlations of root traits with grain yield are given in Supplementary Table 6 under all growing conditions, and per growing condition. The database used for analysis is presented in raw data tables 1, 2, 3, 4 and 5.

Principal component analysis (PCA) of the six selected genotypes grown under the three conditions (INP, RNP, ILP), for both years combined, is displayed in Supplemental Figure 1.

The statistical analysis is based on plot replicates, with each plot presenting a unique value, whether it is a single measurement (e.g. CT and NDVI) or an average of several replicates (e.g. for leaf pigments and root traits, five replicates per plot were assessed). Analytical traits were DTH, by anthesis CT, leaf pigments (chlorophylls, flavonoids, anthocyanins and the nitrogen balance index (NBI)), PH and NDVI. By maturity, GY, HI, TGW, GN, ear density per ear, total grain nitrogen yield (GNY) were assessed, together with root traits assessed (average root width (Width), number of connected components (CComp), and the maximum (MaxR) and median (MedR) number of roots, root network depth (Ndepth), root network length (Nlen), and root network width (Nwidth), network area (NwA), network surface area (Nsurf), and network volume (Nvol), network convex area (ConvA), the ratio of network length to the network volume (specific root length (SRL) the ratio of the maximum root number to the median root number (Network bushiness (Bush)), the total network area divided by the network convex area (Network solidity), the lower 2/3 of the root network depth (length distribution (Ldist)), and the ratio of the network width to the network depth (network width to depth ratio (NWDR), and RA. C and N concentrations on a dry matter basis, and δ^13^C and δ^15^N isotope composition in the dry matter were measured in flag leaves sampled at anthesis and in mature grains, whereas δ^18^O isotope composition in stem water was measured in samples collected at anthesis.

## 2 Experimental Design, Materials and Methods

Field trials were carried out during two consecutive years (2017-2018; 2018-2019) following a complete block design with three replicates. Each plot consisted of seven rows planted 20 cm apart and at a seed rate of 250 seeds m^−2^, representing an area of 7 × 1.5 m^2^. For both crop seasons, a set of 24 post green revolution commercial durum wheat cultivars (*Triticum turgidum* L. subsp. *durum* (Desf) Husn.) were sown under three growing conditions (INP, RNP and ILP).

From among the 24 durum wheat cultivars, six genotypes with contrasting agronomic performance were selected (i.e. high versus low yield). Selection was based on yield data obtained from the previous crop season (2016-2017), in addition to the two crop seasons included in this study (2017-2018 and 2018-2019), and the six genotypes were evaluated at the INIA station as stated above, as well as at a second INIA station located in Coria del Rio (Seville) under irrigated normal planting conditions (Supplemental Table 1).

Measurements were performed at different phenological stages. Around heading, DTH was determined for the first crop season (2017-2018) alone, for each plot, and when approximately 50% of ears had emerged. At anthesis, flag leaves were assessed randomly on the adaxial side of flag leaves from the central rows of each selected plot in all trials and both years, using a leaf pigment meter (Dualex). PH was determined by placing a ruler zenithally in the central rows of each selected plot, then values were taken by observing the whole canopy and averaging the distance from the ground to the overall tip of the ears, excluding the awns. CT was measured with an infrared thermometer, where the IR sensor was placed at a distance of 80 cm from the canopy, pointing the laser beam towards plant leaves with the sun towards the rear [8]. NDVI values were obtained using a portable spectroradiometer (GreenSeeker), by skimming the active sensor perpendicularly across the canopy of each selected plot at a constant height of 60 cm. In the same phenological stage, five different plants were selected in a similar way from the central rows of each plot, and the stem bases from these plants were pooled. Stem bases (approximately 6-7 cm length) were sealed immediately in analytical tubes and frozen at −80 °C until water distillation could be undertaken. At physiological maturity, root traits were assessed in the selected genotypes following the shovelomics method, where five random plants were dug manually from the first 15 cm of soil, and the roots were washed carefully using a hose, then digitized in situ until further analysis. Regarding yield components, ear density (ears m^−2^) was determined by counting the number of ears in a 1 m length of a central row. GN and TGW were assessed using a subset of ten representative plants from the central rows of each plot. HI, which is the ratio of grain weight to total aboveground biomass, was calculated from the same sampled plants. GY was assessed by machine harvesting plots. Mature grains were then collected, dried, ground to a fine powder and analysed for stable isotope composition (δ^13^C and δ^15^N) and elemental analysis (C and N) determination using an elemental analyser coupled with an isotope ratio mass spectrometer (EA-IRMS). For δ^18^O of the stem, xylem distilled water was extracted and the stable isotope composition was determined using a Picarro L2120-I isotopic water analyser coupled to an A0211 high-precision vaporizer.

Raw data were analysed with the SPSS 25 statistical package (IBM SPSS Statistics 25, Inc., Chicago, IL; USA) using a multivariate analysis coupled with a post hoc test (Tukey-b) to assess differences between genotypes within each treatment.

Principal component analyses were used, carried out with the open-source software, RStudio 1.2.5 (R Foundation for Statistical Computing, Vienna, Austria), to analyse all traits in a reduced bi-dimensional platform.

Graphs were created using the SigmaPlot program 10.0 (SPSS Inc.).

## Ethics Statement

The present work did not involve the use of human subjects, animal experiments, or data collected from social media platforms.

## CRediT Author Statement

**Fatima Zahra Rezzouk:** Wrote the first draft, collected samples, conducted the root studies and the stable isotope and statistical analyses, drew the tables and implemented the edits; **José Luis Araus:** participated in the field evaluation, conceived the study and implemented the edits in the consecutive drafts; **Maria Dolores Serret:** collaborated on the stable isotope analyses and revised the drafts; **Adrian Gracia-Romero** and **Shawn C. Kefauver:** ran the remote sensing measurements and collaborated in the shovelomics; **Maria Teresa Nieto-Taladriz:** conducted the field trials and collected the grain yield, the agronomic yield components and the phenological data.

## Declaration of Competing Interest

The authors declare that they have no known competing financial interests or personal relationships that have, or could be perceived to have, influenced the work reported in this article.
